# Supplementary material for: Identification of photoperiod-induced specific miRNAs in the adrenal glands of Sunite sheep (Ovis aries)
Source: Front Vet Sci. 2022 Jul 22;9:888207. doi: 10.3389/fvets.2022.888207 (PMC9354845; doi:10.3389/fvets.2022.888207)
Supplement: Supplementary file 1 [file Data_Sheet_1.ZIP › Supplementary file/miRDeep2 file.zip!/miRDeep2 file/Novel_Structure/SP42A3/Novel_642.pdf]

Provisional ID	:	NC_019460.2_7889
Score total	:	24.6
Score for star read(s)	:	3.9
Score for read counts	:	21.5
Score for mfe	:	-2.3
Score for randfold	:	1.6
Score for cons. seed	:	
Total read count	:	54
Mature read count	:	45
Loop read count	:	0
Star read count	:	9

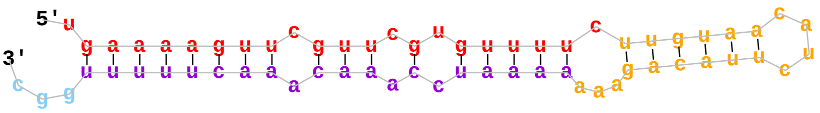

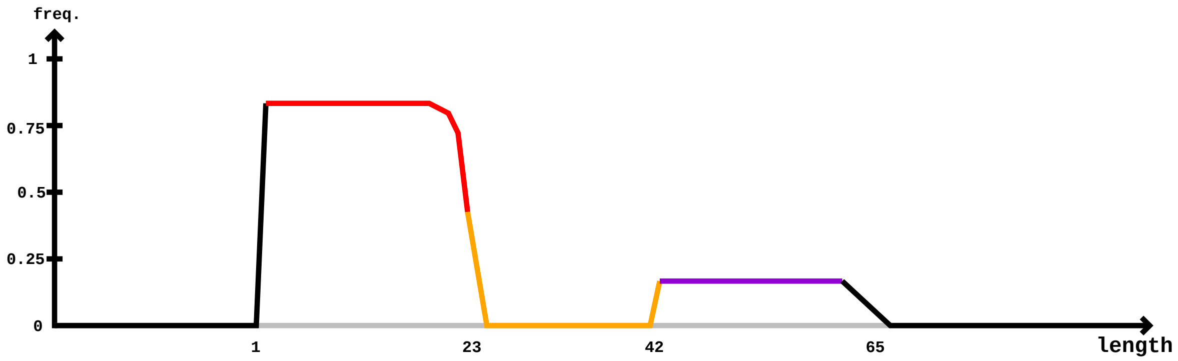

Mature Star

```
5'- cuguaaaggcaacagagggcugaaaaguucguucguguuuucuuguaacaucuuacagaaaaaaauccaaacaaacuuuuuggccaacccaguauauucaacaggacucuug   -3'    obs
    cuguaaaggcaacagagggcugaaaaguucguucguguuuucuuguaacaucuuacagaaaaaaauccaaacaaacuuuuuggccaacccaguauauucaacaggacucuug          exp
    ((((.......))))..((((((((((((.(((.(.(((((.((((((....))))))...))))).).))).))))))))))))...((.((.......)).)).......   reads    mm     sample
    ....................ugaaaaguucguucgugu..........................................................................   1        0      seq
    ....................ugaaaaguucguucguguu.........................................................................   1        0      seq
    ....................ugaaaaguucguucguguuu........................................................................   4        0      seq
    ....................ugaaaaguucguucguguuuu.......................................................................   16       0      seq
    ....................ugaaaaguucguucguguuuuc......................................................................   23       0      seq
    .............................................................aaaauccaaacGaacuuuuu...............................   5        1      seq
    .............................................................aaaauccaaacGaacuuuuug..............................   3        1      seq
    .............................................................aaaauccaaacGaacuuuuugg.............................   1        1      seq
```
